# Depression Associated with Caregiver Quality of Life in Post-COVID-19 Patients in Two Regions of Peru

**DOI:** 10.3390/healthcare10071219

**Published:** 2022-06-29

**Authors:** Janett V. Chávez Sosa, Flor M. Mego Gonzales, Zoila E. Aliaga Ramirez, Mayela Cajachagua Castro, Salomón Huancahuire-Vega

**Affiliations:** 1 Escuela Profesional de Enfermería, Universidad Peruana Unión (UPeU), Lima 15464, Peru; viki16@upeu.edu.pe (J.V.C.S.); flormego@upeu.edu.pe (F.M.M.G.); estheraliaga@upeu.edu.pe (Z.E.A.R.); mayela@upeu.edu.pe (M.C.C.); 2 Escuela Profesional de Medicina, Universidad Peruana Unión (UPeU), Lima 15464, Peru; 3 Dirección General de Investigación, Universidad Peruana Unión (UPeU), Lima 15464, Peru

**Keywords:** depression, quality of life, family caregivers, COVID-19

## Abstract

Due to COVID-19, the workload experienced by caregivers has increased markedly which has led them to experience fatigue, anxiety and depression. This study aims to determine the relationship between quality of life and depression in caregivers of post-COVID-19 patients in two regions of Peru. In a cross-sectional analytical study, the sample was non-probabilistic and by snowball, and consisted of 730 caregivers, to whom the questionnaires “Modified Betty Ferell Quality of Life” and the “Beck Depression Inventory” were applied. It was determined that being a male caregiver (OR: 2.119; 95% CI: 1.332–3.369) was associated with a good quality of life. On the other hand, caregivers who had children (OR: 0.391; 95% CI: 0.227–0.675), were vaccinated against COVID-19 (OR: 0.432; 95% CI: 0.250–0.744), were immediate family members (OR: 0.298; 95% CI: 0.117–0.761) and had high depression (OR: 0.189; 95% CI: 0.073–0.490) were associated with poor quality of life. The results of this study allow us to conclude the association between depression and poor quality of life in caregivers of these patients so it is necessary to monitor the mental health of caregivers, and to develop adaptation strategies to pandemic conditions.

## 1. Introduction

The growing wave of COVID-19 infections caused a major impact worldwide. This crisis led to a state of emergency that triggered radical preventive measures such as confinement, quarantine and social distancing [1]. The contingency measure taken in response to the pandemic was the limitation of health services at the first level of care and other health facilities, as well as the restriction of outpatient activities, health promotion and risk prevention; this lack of access to the health system, hospitals, appointments, beds and oxygen balloons generated the need for people to assume the role of caregivers and care for their family members with COVID-19 at home. In most cases, caregivers were immediate family members such as father, mother, husband, wife, son, daughter, brother, sister, etc. [2].

A study conducted in the United States indicates that there are about 43.5 million informal caregivers caring for sick or disabled family members; these caregivers are untrained, feel unprepared for this role and experience high levels of stress and depression. Lack of preparation and inadequate development of basic skills for quality caregiving affect the caregiver’s emotional well-being, social well-being and finances [3].

At the Latin American level, the workload experienced by caregivers, which includes the parameters of time, difficulty and tasks, is huge and has increased markedly during the pandemic era [4]. According to Ibáñez et al., “the challenges include aspects such as insufficient supply by healthcare personnel, social and socioeconomic determinants of health, disparities, gender-biased burdens and the effect of COVID-19 on families” [5]. Latin American countries face deficiencies in their health systems, which hinder the level of care that the population needs.

Peru has been one of the Latin American countries widely affected by COVID-19, has the highest infection-lethality rate (ILR) due to COVID-19 in the world, and has the fifth highest number of deaths in absolute terms [6]. Even before the pandemic, caregivers of Peruvian patients hospitalized in the medical service were identified as having a poor quality of life [7]. There are different conceptualizations of quality of life related to health. From the nursing point of view, Ferrell et al. describes it as a multidimensional construction that includes well-being or discontent in aspects of life important to the individual, encompassing the interaction of health and psychological, spiritual, socioeconomic and family functioning [8].

Preventive measures taken by the government gave caregivers a new way to manage the prevention of respiratory infections; however, they are not being properly implemented. According to Navarrete et al., the average age of Peruvian caregivers is 33 and 48 years, 95.7% of caregivers in Lima are female, 77.2% of caregivers have not received training on hand washing and 96.7% do not know how to properly manage stress [9].

One of the dimensions of quality of life is spiritual well-being. Spiritual well-being is defined as the satisfaction and experience of a harmonious relationship with oneself/others, nature or a power greater than oneself [10]. Different studies show the importance of spiritual well-being during the COVID-19 pandemic, both for patients with COVID-19 and their caregivers [11,12]. Psychological well-being is another component of quality of life. This is defined by the WHO as the state of complete physical, mental and social well-being, and not merely the absence of disease or illness [13]. There is evidence showing that psychological well-being is positively related to behaviors related to health and spiritual aspects [14].

Another factor affecting quality of life is depression. Depression is a frequently occurring disorder that affects the ability to perform day-to-day tasks effectively and makes it difficult to cope adequately with problems [15]. Therefore, depression is one of the most present and prevalent problems in caregivers because it is directly related to caregiver overload and quality of life; it mainly results from a lack of organization and care by the healthcare system, the absence of a solid support network for the caregiver and insufficient experience in caring for the patient [16]. The Beck Depression Inventory I is one of the most widely used scales in the world to measure the severity of depression symptoms in clinical and non-clinical samples. Based on this scale in its Spanish version, we consider “high depression” scores above 14 [17].

Studies prior to the pandemic found that the frequency of anxiety and depression in primary caregivers of pediatric patients was higher than in the general population [18]. Likewise, in another study, it was found that the quality of life in elderly caregivers was worse compared to younger caregivers due to their frailty, which leads many of them to experience symptoms of depression and a poorer quality of life [19].

There is a variety of research on caregiver quality of life; however, little research has been conducted on the quality of life of caregivers of post-COVID-19 patients. The work performed by the caregiver is of vital importance in the midst of the disease and in the patient’s recovery. However, it has a stressful effect on oneself because of the feeling of constant helplessness, the uncertain future and fatigue, which is mainly related to depressive symptomatology and other health conditions in the physical, social, spiritual and occupational areas [20]. As a result, conflicts in the different areas of the caregiver’s life generate changes in the family relationship; altering its correct functioning which is expressed through the ability to resolve conflicts, family crises and at the moment of expressing affection and care [21].

Therefore, the study aimed to determine the relationship between quality of life, including physical and psychological well-being, social welfare and spiritual wellness with depression in caregivers of post-COVID-19 patients in two regions of Peru during the second wave of the pandemic.

## 2. Materials and Methods

The study was a cross-sectional analytical study. The sampling was non-probabilistic and by snowball, and included caregivers of both sexes, over 18 years of age, who were caring for family members or patients diagnosed with COVID-19 and who agreed to participate voluntarily in the study. The sample consisted of 730 caregivers, of whom 366 were from the east of Lima (capital, cost) and 364 from Pucallpa-Ucayali (eastern of Peru, jungle).

For the measurement of quality of life, the modified Betty Ferrell quality of life scale was used, with a reliability of 0.88 by Cronbach’s alpha [22]. It has 30 items distributed in four dimensions: physical, psychological, social and spiritual well-being, with a Likert-type response scale. The following classification was used to measure the results of the instrument: 54 to 84 points is equivalent to a poor quality of life and 85 to 115 points as good quality. On the other hand, the depression variable was measured with the Beck Depression Inventory, with a reliability of 0.91 by Cronbach’s alpha. This instrument is composed of 21 items, with a Likert-type response scale. The final score ranges from 0 to 13 points equivalent to low depression and from 14 to 35 points as high depression [17].

To collect the data, a letter of request for authorization was first presented to the authorities. For data collection, the survey technique was used, through home visits in the study areas and respecting the biosecurity protocols against COVID-19 imposed by the government. The study has the approval of the Ethics Committee of Peruvian Union University (N° 2021-CE-FCS-UPeU-016) and informed consent was obtained before the application of the questionnaire.

Data analysis was performed using the SPSS v.24 statistical program. For univariate analysis, simple frequency tables were used, and for bivariate analysis, contingency tables and the chi-square test were used. For multivariate analysis, binary logistic regression was used, considering quality of life as the dependent variable: poor (0) and good (1). Similarly, sociodemographic characteristics and depression in caregivers of post-COVID-19 patients were considered as independent variables.

## 3. Results

Of 730 caregivers, 76.3% were women and 23.7% were men. Likewise, 58.2% were aged 30–59 years, 50.8% were from Lima, 56% were married or cohabiting and 74% had higher education. On the other hand, 83.6% were employed, 46.3% had EsSalud insurance (social security in the country), 84.8% had already been vaccinated against COVID-19, 56.2% had children and 96.4% were direct relatives of the post-COVID-19 patient (Table 1).

Regarding the study variables, 80.1% of the caregivers presented poor quality of life, as did the psychological and spiritual well-being dimensions, with 82.7% and 67.1%, respectively. On the other hand, 86.4% of caregivers had low depression and 13.6% had high depression. The same trend is observed for each of its dimensions (Table 2).

Bivariate analysis showed that sex (*p* = 0.000), age (*p* = 0.001), place of origin (*p* = 0.006), marital status (*p* = 0.000), educational level (*p* = 0.017), type of work (*p* = 0.000), health insurance (*p* = 0.009), COVID-19 vaccination (*p* = 0.000), children (*p* = 0.000), relationship to the patient (*p* = 0.044), and depression were related to caregivers’ quality of life (Table 3).

Finally, multivariate analysis found that being a male caregiver (OR: 2.119; 95% CI: 1.332–3.369) was associated with good quality of life. On the other hand, caregivers who had children (OR: 0.391; 95% CI: 0.227–0.675), were vaccinated against COVID-19 (OR: 0.432; 95% CI: 0.250–0.744), were immediate family members (OR: 0.298; 95% CI: 0.117–0.761) and had high depression (OR: 0.189; 95% CI: 0.073–0.490) were associated with poor quality of life (Table 4).

## 4. Discussion

In the literature, there are studies that indicate that the quality of life of caregivers is slightly or moderately related to the state of mental, physical and social health. On the other hand, Troschel et al. mentions that the quality of life of caregivers can be significantly reduced if the emotional burden increases [23]. In this work with caregivers of post-COVID-19 patients, it was evidenced that the caregivers presented poor quality of life, especially in the dimensions of psychological and spiritual well-being and it was determined that quality of life is strongly associated with depression, in addition to other factors such as male sex, having children and being a direct relative.

Recent studies have shown lower levels of spiritual well-being than the situation prior to the pandemic, in addition, it has been shown that caregivers of COVID-19 patients express spiritual needs and that they use spiritual coping strategies [24]. During critical health circumstances such as the recent pandemic, many caregivers have questioned the meaning of life and recognized hopelessness, taking refuge in the sacred and using religious practices in search of spiritual well-being [25]. It would be necessary to consider the spiritual aspects of each individual as an important resource in obtaining general well-being.

In relation to the characteristics of the caregivers analyzed in this study, similarities were found with what is described in other investigations [26]. In the study population, the role of caregiver is mainly assumed by women, with 76.3% of the respondents belonging to the female sex. There are also other studies that demonstrate the high prevalence of the female sex as the main caregiver, demonstrated in a study applied to caregivers in China finding that 62.65% of the caregivers were women [27], as well as that of Ito and Tadaka in their work on the Japanese population, where 79.7% of family caregivers were female [28].

This study found that female caregivers had poorer quality of life than male caregivers. The literature indicates that female caregivers are more likely to have higher anxiety and depression and lower social support than male caregivers, which negatively impacts their quality of life [29]. In terms of caregiving approach, male caregivers have a more task-oriented approach to caregiving, while female caregivers use more emotion-oriented coping methods. Additionally, female caregivers tend to report a higher degree of caregiver burden and psychological distress; however, male caregivers may be reluctant to disclose feelings of burden or distress due to traditional views of masculinity that idealize self-sufficiency and stoicism [30].

Next, 56.2% of the caregivers claimed to have children and this was associated with a poor quality of life. In this sense, Pucciarelli, in his study, found that the quality of life of younger family caregivers without children was higher than that of those who had children [31]. The literature indicates that younger mothers perceive a greater burden with each additional child they have than do older mothers [32]. Likewise, each child increases the family burden and this would be related to a poorer quality of life [33].

It was also shown that caregivers who were vaccinated had a poor quality of life. In contrast, a study in the Polish population revealed that fully vaccinated persons had lower levels of anxiety, better quality of life and lower subjective anxiety about being infected with COVID-19 than those awaiting vaccination or those with an incomplete (one dose) vaccination regimen [34]. However, the review of the literature indicates that most of the population presents a local or systemic reaction after the application of the vaccine against COVID-19 and that these reactions could generate work limitation after the application of the second dose, which could affect their quality of life [35,36].

On the other hand, caregivers who were direct relatives of the post-COVID-19 patient presented worse quality of life. Previous studies revealed that family caregivers presented a poor quality of life due to low preparedness for patient care [37]. Likewise, spouses of palliative care partners reported poorer quality of life due to higher levels of unmet support needs [38].

Regarding depression, our study found that 86.4% of caregivers had low depression. Several studies show that caregivers of patients have high percentages of mild to moderate depression [39,40]. In addition, the study found that the higher the level of depression, the poorer the caregiver’s quality of life. Similarly, a study of primary caregivers of children with cerebral palsy showed that greater severity of depression is negatively associated with dimensions of quality of life [41]. In the same way, a systematic review revealed that caregivers of patients with Dravet syndrome have high levels of depression and anxiety and that they are associated with fatigue and poor sleep quality, which affected their quality of life [42]. Finally, a study in caregivers of patients with heart failure also showed that depression and anxiety were associated with fatigue and poor sleep quality, which affected their quality of life [43]. These facts indicate that poor mental health, such as the presence of high levels of depression, impairs quality of life conditions. The COVID-19 pandemic has affected the mental health of the population, with several studies showing increased levels of depression [44,45]. Caregivers of post-COVID-19 patients are also included, who in addition to their work as caregivers must face the fear of contagion and the disease. These conditions would be affecting their quality of life, so it is necessary to monitor the mental health of this population group.

The study has some limitations, the main one being that it was carried out in populations of two regions of the country, the capital and the jungle region; it was not carried out in the highlands, so the results cannot be generalized to the entire Peruvian population. In addition, it is a cross-sectional study, so causality cannot be determined. Finally, some factors that could influence the quality of life of caregivers such as caregiver overload and preparation and other mental health conditions such as fear, worry and anxiety were not considered. Despite these limitations, it is necessary to highlight that to our knowledge this is the first report on the quality of life conditions of caregivers of post-COVID-19 patients in the Peruvian population.

## 5. Conclusions

The recovery process of post-COVID-19 patients depends to a great extent on the work of their caregivers, therefore, their self-care and quality of life should be promoted, including spiritual and psychological well-being. The results of this study allow us to conclude the association between depression and poor quality of life in caregivers of these patients and, in addition, it has been found that having children, having received the vaccine and being a direct relative are associated with a good quality of life. It is necessary to monitor the mental health of caregivers and to develop adaptation strategies to pandemic conditions and, besides, it would be advisable to assign fewer hours of work and greater inclusion in social activities. This will be reflected in better care and recovery of patients post COVID-19.

## Figures and Tables

**Table 1:** General characteristics of caregivers of post-COVID-19 patients.

Variables		*n* = 730	%
Sex	Male	173	23.7
	Female	557	76.3
Age	Young (18–29 years old)	305	41.8
	Adult (30–59 years)	425	58.2
City	Lima	371	50.8
	Pucallpa-Ucayali	359	49.2
Marital status	Single/Widowed/Divorced	321	44
	Married/Cohabitant	409	56
Level of education	Basic education	190	26
	Higher education	540	74
Type of work	Not working/Retired	120	16.4
	Dependent/Independent	610	83.6
Type of insurance	No insurance	15	2.1
	SIS	286	39.2
	ESSALUD	338	46.3
	PNP/FFAA	11	1.5
	Private	80	11
COVID-19 vaccination	Yes	619	84.8
	No/Not my turn yet	111	15.2
Do you have children?	Yes	410	56.2
	No	320	43.8
Relationship to patient	Immediate family member	704	96.4
	Non-direct relative	26	3.6

**Table 2:** Descriptive analysis of the study variables.

Variables		*n* = 730	%
Quality of Life	Good	145	19.9
	Deficient	585	80.1
Physical Well-being	Good	424	58.1
	Deficient	306	41.9
Psychological Well-being	Good	126	17.3
	Deficient	604	82.7
Social Welfare	Good	389	53.3
	Deficient	341	46.7
Spiritual Wellness	Good	240	32.9
	Deficient	490	67.1
Depression	High	99	13.6
	Low	631	86.4
Cognitive Area	High	25	3.4
	Low	705	96.6
Physical Behavioral Area	High	51	7
	Low	679	93
Affective Emotional Area	High	103	14.1
	Low	627	85.9

**Table 3:** Bivariate analysis according to caregiver quality of life post COVID-19.

Variable		Quality of Life				*p*-Value
		Good		Deficient		
		*n*	%	*n*	%	
Sex	Female	95	65.5	462	79	0.001 *
	Male	50	34.5	123	21	
Age	Young (18–29 years old)	90	62.1	215	36.8	0.000 *
	Adult (30–59 years old)	55	37.9	370	63.2	
City	Lima	59	40.7	312	53.3	0.006 *
	Pucallpa-Ucayali	86	59.3	273	46.7	
Marital status	Single/Widowed/Divorced	92	63.4	229	39.1	0.000 *
	Married/Cohabitant	53	36.6	356	60.9	
Do you have children?	No	100	69	220	37.6	0.000 *
	Yes	45	31	365	62.4	
Studies	Basic education	49	33.8	141	24.1	0.017 *
	Higher education	96	66.2	444	75.9	
Do you have a job?	No	42	29	78	13.3	0.000 *
	Yes	103	71	507	86.7	
Do you have health insurance?	No	7	4.8	8	1.4	0.009 *
	Yes	138	95.2	577	98.6	
Have you been vaccinated against COVID-19?	No	49	33.8	62	10.6	0.000 *
	Yes	96	66.2	523	89.4	
Relationship to patient	Immediate family member	136	93.8	568	97.1	0.044 *
	Non-direct relative	9	6.2	17	2.9	
Depression	High	5	3.4	94	16.1	0.000 *
	Low	140	96.6	491	83.9	

* Statistical significance *p* < 0.05.

**Table 4:** Multivariate analysis according to the quality of life of the caregiver of post-COVID-19 patients.

Variables		OR	95% CI		*p*-Value
			LI	LS	
Sex	Male	1	(Reference)		
	Female	2.119	1.332	3.369	0.002 *
Age	Young (18–29 years old)	1	(Reference)		
	Adult (30–59 years)	0.860	0.491	1.507	0.599
City	Lima	1	(Reference)		
	Pucallpa-Ucayali	1.356	0.883	2.084	0.164
Marital status	Single/Widowed/Divorced	1	(Reference)		
	Married/Cohabitant	0.874	0.499	1.530	0.637
Do you have children?	No	1	(Reference)		
	Yes	0.391	0.227	0.675	0.001 *
Studies	Basic education	1	(Reference)		
	Higher education	1.001	0.608	1.647	0.998
Do you have a job?	No	1	(Reference)		
	Yes	0.665	0.383	1.152	0.146
Do you have health insurance?	No	1	(Reference)		
	Yes	0.696	0.214	2.263	0.547
Have you been vaccinated against COVID-19?	No	1	(Reference)		
	Yes	0.432	0.250	0.744	0.002 *
Relationship to patient	Immediate family member	1	(Reference)		
	Non-direct relative	0.298	0.117	0.761	0.011 *
Depression	High	1	(Reference)		
	Low	0.189	0.073	0.490	0.001 *

* Statistical significance *p* < 0.05; LI: lower limit; LS: upper limit.

## Data Availability

The data presented in this study are available by request to the lead author.
